# Initiation and continuity of maternal healthcare: examining the role of vouchers and user-fee removal on maternal health service use in Kenya

**DOI:** 10.1093/heapol/czz004

**Published:** 2019-03-06

**Authors:** Mardieh L Dennis, Lenka Benova, Timothy Abuya, Matteo Quartagno, Ben Bellows, Oona M R Campbell

**Affiliations:** 1 Department of Infectious Disease Epidemiology, Faculty of Epidemiology & Population Health, London School of Hygiene and Tropical Medicine, Keppel Street WC1E7HT, London, UK; 2 Department of Public Health, Institute of Tropical Medicine, Kronenburgstraat 43, Antwerpen, Belgium; 3 Population Council Kenya, Avenue 5, Rose Avenue, Nairobi, Kenya; 4 Department of Medical Statistics, Faculty of Epidemiology & Population Health, London School of Hygiene and Tropical Medicine, Keppel Street WC1E7HT, London, UK; 5 MRC Clinical Trials Unit, Institute of Clinical Trials and Methodology, University College London, Gower Street, London, UK and; 6 Population Council, 4301 Connecticut Avenue NW, Suite 280, Washington DC, USA

**Keywords:** User fees, vouchers, maternal health, private sector, Kenya, continuum of care

## Abstract

This study explores the relationship between two health financing initiatives on women’s progression through the maternal health continuum in Kenya: a subsidized reproductive health voucher programme (2006–16) and the introduction of free maternity services in all government facilities (2013). Using cross-sectional survey data, we ran three multivariable logistic regression models examining the effects of the voucher programme, free maternity policy, health insurance and other determinants on (1) early antenatal care (ANC) initiation (first visit within the first trimester of pregnancy), (2) receiving continuous care (1+ ANC, facility birth, 1+ post-natal care (PNC) check) and (3) completing the maternal health pathway as recommended (4+ ANC, facility birth, 1+ PNC, with first check occurring within 48 h of delivery). Full implementation of the voucher programme was positively associated with receiving continuous care among users of 1+ ANC [interaction term adjusted odds ratio (aOR): 1.33, *P* = 0.014]. Early ANC initiation (aOR: 1.32, *P* = 0.001) and use of private sector ANC (aOR: 1.93, *P* < 0.001) were also positively associated with use of continuous care among ANC users. Among continuous care users, early ANC was associated with increased odds of completing the maternal health pathway as recommended (aOR: 3.80, *P* < 0.001). Higher parity was negatively associated with all three outcomes, while having health insurance was positively associated with each outcome. The impact of other sociodemographic factors such as maternal age, education, wealth quintile, urban residence, and employment varied by outcome; however, the findings generally suggest that marginalized women faced greater barriers to early ANC initiation and continuity of care. Health financing and women’s timing and source of ANC are strongly related to their subsequent progression through the maternal health pathway. To increase continuity of care and improve maternal health outcomes, policymakers must therefore focus on equitably reducing financial and other barriers to care seeking and improving quality of care throughout the continuum.

**Key Messages**

- There is insufficient evidence on the impact of health financing strategies on continuum of maternal healthcare from the first antenatal care (ANC) visit to post-natal care in low- and middle-income countries.
- Our study in Kenya found that access to subsidized maternal health vouchers and health insurance were associated with improved continuity of care; however, socio-economic disparities in access to care persisted after controlling for access to various health financing strategies.
- To improve timely ANC initiation and retention of women in the maternal health service continuum, policymakers must focus not only on optimizing health financing schemes to equitably reduce financial barriers to care seeking, but also on reducing non-financial barriers and improving quality of care throughout the continuum.

## Introduction

From 1990 to 2015, the global maternal mortality ratio (MMR) decreased by 44% from an estimated 385 to 216 maternal deaths per 100 000 live births (Alkema *et al.*, 2016). Over the same period, Kenya’s MMR decreased by only 26% from 687 to 510; this is below both the average global decline and the country’s Millennium Development Goal 5a target of a 75% reduction (United Nations, 2015). Kenya’s comparatively slow reduction in maternal mortality is likely due to insufficient coverage of maternal health services; for instance, in 2014, an estimated 58% of women in Kenya attended at least four antenatal care (ANC) visits, 62% gave birth with the assistance of a skilled birth attendant, and 57% received a post-natal care (PNC) check (Kenya National Bureau of Statistics *et al.*, 2015). As ability to pay remains an important determinant of women’s access to healthcare, many countries have sought to improve coverage of maternal services by reducing financial barriers to service seeking (Gabrysch and Campbell, 2009; Dzakpasu *et al.*, 2014). Strategies implemented at the country level include national health insurance and user fee removals/exemptions, and at the subnational level, community-based health insurance, health vouchers and conditional cash transfers (Ensor and Ronoh, 2005).

Global development organizations and policymakers argue that continuity of care throughout the antenatal, intra-partum and post-partum periods is essential for improved maternal health outcomes (Kerber *et al.*, 2007; UNICEF and World Health Organization, 2015; World Health Organization, 2017). Although it is recommended for women to receive all of these services for each pregnancy, efforts to monitor progress towards global development goals have tended to track coverage indicators in a cross-sectional nature by service type rather than tracking indicators of continuity of care longitudinally for each birth (World Health Organization, 2005; UNICEF and World Health Organization, 2015). Similarly, the effects of maternal health financing strategies globally and in Kenya have been assessed by examining use of care at individual points along the maternal health continuum. While many of these studies suggest that vouchers, health insurance, and reducing or eliminating user fees increase coverage of ANC, facility delivery and PNC individually, there has been no focus on how such financing mechanisms affect continuity of maternal care as measured from the perspective of women’s pathways from pregnancy to the post-partum period (Bellows *et al.*, 2011; Brody *et al.*, 2013; Comfort *et al.*, 2013; Dzakpasu *et al.*, 2014; Gopalan *et al.*, 2014; Wang *et al.*, 2016; Hunter *et al.*, 2017).

With funding from the German Development Bank (KfW), the Kenyan Ministry of Health and partners implemented a reproductive health voucher programme from 2006 to 2016, aimed at reducing inequitable access to maternal care (Abuya *et al.*, 2012). Under this programme, poor women could purchase subsidized vouchers for 200 Kenyan Shillings (≈$2.20) that covered the cost of four ANC visits, facility delivery (vaginal or caesarean) and PNC. In order to be accredited for participation in the programme, health facilities were required to meet minimum quality standards based on national guidelines for the provision of maternal care. Women could redeem vouchers at any participating public or private sector facility, and the contracted facilities submitted claims to be reimbursed at standard rates for each service provided. In June 2013, 7 years after the start of the voucher programme, the Kenyan government announced the inception of the free maternity services policy, which called for all public health facilities to provide maternal health services at no cost to users. While some facilities interpreted the policy to include all services across the maternal health continuum, others offered delivery care for free and continued to charge for ANC and/or PNC (Pyone *et al.*, 2017). Similar to the voucher programme, public facilities were to be reimbursed for each client served under the free maternity services policy; however, many facilities reported challenges and delays in receiving these reimbursements (Tama *et al.*, 2017; Abuya *et al.*, 2018).

Given that the voucher programme and free maternity services policy in Kenya targeted key services in the maternal health continuum, they provide a unique setting in which to assess how these two different mechanisms of lowering financial barriers affected women’s continuity of care. In a previous paper, we demonstrated that both the voucher programme and free maternity services policy in Kenya increased women’s use of facilities for childbirth in our study population; however, neither intervention appeared to impact use of 4+ ANC or PNC individually (Dennis *et al.*, 2018). Additionally, we found that while coverage of each individual service was above 60% after the introduction of free maternity services, the use of the recommended maternal care package (defined as 4+ ANC visits initiated within the first trimester, facility delivery and PNC within 48 h of delivery) remained below 25% in both voucher and comparison counties. This article aims to build upon these findings by describing women’s progression through the maternal health continuum and examining the effects of the voucher programme, free maternity services policy, health insurance and other determinants of continuity of care. Specifically, we seek to answer the following questions: (1) what are the determinants of how early a woman initiates ANC during her pregnancy; (2) among women with at least one ANC visit, what are factors influencing subsequent use of both facility delivery and PNC; and (3) among women who receive ANC, facility delivery and PNC, what determines whether they receive all three services at the recommended ANC intensity and PNC timing?

## Methods

### Sampling and data collection

As described previously, this study uses data from three cross-sectional household surveys completed in 2011, 2012 and 2016 (Obare *et al.*, 2013; Dennis *et al.*, 2018). Seven counties were surveyed: four participating in the voucher programme (intervention counties: Kiambu, Kilifi, Kisumu and Kitui) and three where vouchers were not provided (comparison counties: Makueni, Nyandarua and Uasin Gishu). Comparison counties were matched to the intervention counties based on geographic location, population characteristics and availability of similar health facilities. One intervention county (Kilifi) was not surveyed in 2016 and was therefore excluded from this analysis.

The target sample size within each county was 400 women and these participants were identified using a multi-stage sampling process. County sub-locations within 5 km of a voucher programme accredited facility or similar facility in a comparison county formed the sampling frame for this study. In stage one, 14 sub-locations within each county were randomly selected among those within a 5-km radius of an eligible facility. Three villages were randomly selected from each sub-location in the second sampling stage. Within each village, the poorest households were identified with assistance from local administrators and selected for inclusion in the study, based on their responses to a poverty assessment tool. This purposive sampling was done to ensure that the study sample was predominantly poor, as the voucher programme intended to target poor women. Women aged 15–49 years who were pregnant or reported at least one birth in the past 12 months were invited to participate in the study. In households with more than one woman meeting the study inclusion criteria, the youngest eligible woman was selected for participation.

The interviews covered topics related to women’s household characteristics, reproductive history and use of family planning and reproductive health services. Participants’ responses were recorded by trained interviewers into a tablet-based questionnaire.

### Study population

Responses from all women aged 15–49 years who reported at least one live birth in the 5 years preceding the survey were included in this analysis. We conducted analyses among all births reported in the past 5 years. Supplementary data 1 contains a table with background characteristics of the women included in the sample. Additionally, to better contextualize the wealth distribution of the women included in our sample relative to that of the total population, we described the distribution of selected household assets by wealth quintile in the 2014 Kenya Demographic Health Survey and in the voucher study surveys (Supplementary data 2).

### Indicators and definitions

#### Study periods

Births were categorized into three periods according to when they occurred. Period 1 refers to the pre-intervention and rollout phase of the voucher programme (May 2005 to December 2009). Period 2 refers to the phase during which the voucher programme was fully implemented in all intervention counties and before the free maternity services programme was introduced (January 2010 to May 2013). Finally, Period 3 refers to the phase after the free maternity services programme was introduced in both intervention and comparison counties during which the voucher programme was also fully implemented in all intervention counties (June 2013 to August 2016).

#### Maternal health service coverage and sector of care

We defined the maternal health service use indicators as described in Table 1. For ANC, we defined intensity of care in terms of the number of ANC visits received and the timing of ANC initiation (early vs delayed). As both the voucher programme and free maternity services policy aimed to encourage women to give birth in health facilities, we defined delivery care in terms of whether a woman delivered in a health facility. For PNC, we considered women who reported receiving a check on their health after delivery to have received PNC. Among those who received PNC, we examined the timing of the first check after birth (timely vs delayed).

**Table 1.** Use of care across the maternal health continuum among all births

Indicator	Definition
Antenatal care (ANC)	
1+ ANC	Received one or more ANC visits; all other births were classified as receiving no ANC
4+ ANC	Received four or more ANC visits
Early ANC	Initiated ANC within the first 3 months (first trimester) of pregnancy
Delayed ANC	Initiated ANC in the fourth month of pregnancy or later
Delivery care	
Facility delivery	Birth that occurred in a health facility; all other births (e.g. those that occurred at home or in another non-facility location) were classified as not being a facility delivery
Post-natal care (PNC)	
Received PNC	Health worker checked on the mother’s health after giving birth; births for which a health worker checked on the baby’s health but not on the woman’s health were classified as having not received PNC
Timely PNC	PNC users who received their first PNC check within 48 h of delivery
Delayed PNC	PNC users who received their first PNC check more than 48 h after delivery
Continuum of maternal care (among users of 1+ ANC)	
Discontinuous care	Received at least one service (ANC, facility delivery, or PNC) during the maternal period, but did not receive all three services
Continuous care, suboptimal	Made contact with health services during each point of the maternal health continuum (received 1+ ANC visit, facility delivery and PNC), but did not receive care at the recommended ANC intensity (4+ ANC) and /or PNC timing (within 48 h of birth), irrespective of ANC initiation timing
Continuous care, completed pathway	Received 4+ ANC, facility delivery and PNC within 48 h of delivery were classified as having received continuous care and completed the continuum of maternal care pathway, irrespective ANC initiation timing
Sector of care (among continuous care users—both suboptimal and completed pathway)	
Public sector	Received ANC, facility delivery and PNC entirely in the public sector; a small proportion of continuous care users (<1%) who received either ANC and/or PNC at home, and facility delivery in the public sector, were also classified as having received public sector care
Private sector	Received ANC, facility delivery and PNC entirely in the private sector (including for profit, not-for-profit and faith-based)
Mixed, public and private sector	Received ANC, facility delivery and PNC from at least one public sector source and at least one private sector source

We also report on indicators related to use of all three health services across the maternal health continuum (Table 1). We examined women’s progression through the continuum of care among 1+ ANC users grouped into three categories: (1) discontinuous, (2) continuous, suboptimal care and (3) continuous care, completed pathway; these categories are mutually exclusive (Table 2). As our interest was in women’s continuity of care after making contact with the health system through their first ANC visit, these definitions do not take into account ANC timing. Instead, we examined the timing of ANC initiation as a determinant of continuity of care.

**Table 2.** Continuity of care classifications

	1+ ANC	4+ ANC	Facility delivery	PNC	PNC within 48 hours
Discontinuous care					
1+ ANC only	Yes	No	No	No	No
4+ ANC only	Yes	Yes	No	No	No
1+ ANC and facility delivery	Yes	No	Yes	No	No
4+ ANC and facility delivery	Yes	Yes	Yes	No	No
1+ ANC and delayed PNC	Yes	No	No	Yes	No
4+ ANC and delayed PNC	Yes	Yes	No	Yes	No
1+ ANC and timely PNC	Yes	No	No	Yes	Yes
4+ ANC and timely PNC	Yes	Yes	No	Yes	Yes
Continuous, suboptimal care					
1+ ANC and facility delivery and delayed PNC	Yes	No	Yes	Yes	No
4+ ANC and facility delivery and delayed PNC	Yes	Yes	Yes	Yes	No
1+ ANC and facility delivery and timely PNC	Yes	No	Yes	Yes	Yes
Continuous, completed pathway					
4+ ANC and facility delivery and timely PNC	Yes	Yes	Yes	Yes	Yes

### Data analysis

All analyses were conducted at the population level; as such, the intervention groups in this study compared counties exposed to the voucher programme (voucher counties) to those not exposed to the programme (comparison counties) rather than voucher users to non-users.

**Figure 1.**
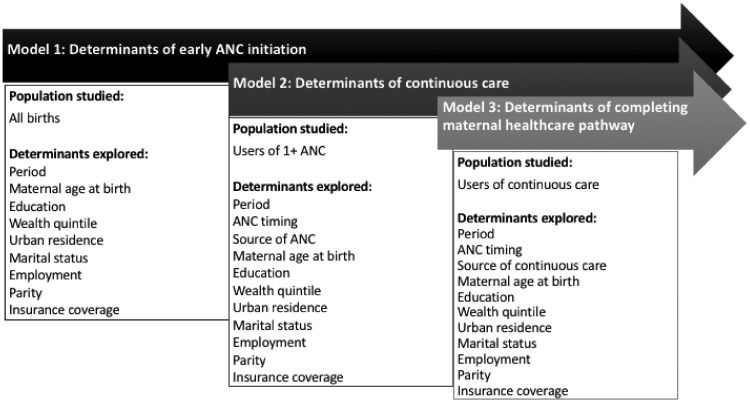
Diagram of three-step logistic analysis approach.

We ran a series of three multivariable logistic regression models to explore the determinants of (1) early ANC initiation among all births, (2) receipt of continuous care among 1+ ANC users, and (3) completing the maternal health pathway among continuous care users (Figure 1). We examined drivers of early ANC initiation based on the assumption that ANC timing is a key determinant of completing the maternal health pathway as recommended. As use of 1+ ANC was nearly universal—above 95% across intervention groups and period—we did not explore determinants of using ANC. For each model, we examined changes over time and the relationship between women’s background characteristics (maternal age at birth, education, wealth quintile, residence, marital status, employment, parity and insurance coverage) and our outcomes of interest. We also explored the effects of ANC timing and source of care as determinants of continuity of care in models examining use of continuous care and completing the maternal health pathway as recommended. We included an interaction term between intervention group and period to assess the impact of the voucher programme on our outcomes of interest. All regression models were adjusted for year of birth and clustering at the county sub-location, village, and woman level, as some women reported multiple live births within the survey recall period.

Due to an error in the tablet-based questionnaire programming for the 2016 survey, 23% of women with one or more births had a missing response for the question on their number of births in the past 5 years. Women missing information on this variable were not asked questions related to maternal health service use; we are therefore missing information on the study outcomes for these women. Due to the nature of the missing data mechanism, we have assumed these data to be missing at random and conducted a complete case analysis. Our analysis of the missing data in the 2016 survey is described in more detail elsewhere (Dennis *et al.*, 2018). Similarly, in the 2011 and 2012 surveys, a small subset of women have complete information for ANC but are missing information on delivery care and PNC due to an input error which caused the survey programme to skip the delivery care and PNC modules. We have assumed these data to be missing at random given year of birth and conducted a complete case analysis, adjusting for year of birth in all inferential analyses. As the input errors resulted in missing data for <5% of all births reported in the 2011 and 2012 surveys, we believe that the impact of this loss of data on our analyses is likely to be negligible. All other variables in this analysis had <1% of responses missing.

All analyses were conducted using Stata IC version 15.1 (StataCorp LLC).

## Results

### Use and timing of ANC

In both voucher and comparison counties, >95% of births received 1+ ANC visits across all three periods; however, most ANC users had a delayed first visit, occurring after the first trimester of pregnancy (Table 3). While approximately 20% of births in Periods 1 and 2 used ANC and initiated ANC early in both study groups, by Period 3, nearly one-third of women in voucher counties started ANC early compared with one-fourth of women in comparison counties.

**Table 3.** Use of care across the maternal health continuum among all births, column percentages

	Comparison counties			Voucher counties		
	Period 1	Period 2	Period 3	Period 1	Period 2	Period 3
Use and timing of ANC						
No ANC	1.4%	2.5%	2.3%	1.4%	3.3%	1.5%
1+ ANC: Delayed ANC	80.0%	78.4%	74.4%	79.5%	75.1%	64.8%
1+ ANC: Early ANC	18.6%	19.1%	23.3%	19.1%	21.6%	32.7%
Total no. of births	1489	1269	641	1672	1344	721
Use of care across the continuum among all users of 1+ ANC						
Discontinuous care	52.2%	47.8%	30.3%	52.0%	38.9%	23.0%
Continuous care (suboptimal)	17.2%	18.7%	22.9%	16.3%	22.4%	20.9%
Continuous care (completed pathway)	30.6%	33.5%	46.8%	31.7%	38.7%	56.1%
Total no. of ANC users	1382	1200	621	1558	1258	703

With regards to determinants of ANC timing, we found that higher parity was associated with reduced odds of early ANC initiation (Table 4). The odds of starting ANC within the first trimester were 44% lower [adjusted odds ratio (aOR) = 0.56; 95% confidence interval (CI): 0.43–0.73] among births to mothers with four or more children and 25% lower (aOR = 0.75; 95% CI: 0.62–0.89) among births to mothers with two to three children compared with women pregnant with their first births. Maternal age 35 years and older (aOR = 0.75; 95% CI: 0.56–1.00) and urban residence (aOR = 0.78; 95% CI: 0.62–0.98) also appear to be associated with later ANC initiation. Women with health insurance coverage had 1.29 times greater adjusted odds of initiating ANC within the first trimester of their pregnancy (95% CI: 1.06–1.58). Belonging to the least poor wealth quintile (aOR = 1.31; 95% CI: 1.03–1.67) and being currently married (aOR = 1.22; 95% CI: 1.02–1.45) were also associated with early ANC initiation.

**Table 4:** Model 1—determinants of early ANC among all births (*n* = 7136)

	Unadjusted		Adjusted^b^	
	OR^a^ [95% CI]	Wald test (*P*-value)	aOR^a^ [95% CI]	Wald test (*P*-value)
Intervention group				
Comparison county	Reference		Reference	
Voucher county	1.21 [1.02, 1.42]	0.025	1.21 [0.95, 1.54]	0.126
Period				
Period 2 (base = Period 1)	1.13 [0.99, 1.29]	0.079	1.23 [0.51, 2.97]	0.641
Period 3 (base = Period 2)	1.52 [1.27, 1.82]	<0.001	1.08 [0.61, 1.91]	0.775
Interaction terms				
Period 2 × Voucher county	1.14 [0.87, 1.50]	0.345	1.12 [0.85, 1.49]	0.408
Period 3 × Voucher county	1.35 [0.95, 1.92]	0.097	1.35 [0.95, 1.93]	0.097
Maternal age at birth				
<25 years	Reference		Reference	
25–34 years	0.83 [0.73, 0.95]	0.008	0.96 [0.82, 1.14]	0.656
≥35 years	0.55 [0.44, 0.67]	<0.001	0.75 [0.56, 1.00]	0.051
Highest level of education				
No education and incomplete primary	Reference		Reference	
Completed primary and incomplete secondary	0.99 [0.86, 1.15]	0.939	0.86 [0.74, 1.00]	0.057
Completed secondary/higher	1.40 [1.15, 1.71]	0.001	0.99 [0.80, 1.24]	0.958
Wealth quintile				
Poorest	Reference		Reference	
Poorer	0.97 [0.82, 1.15]	0.761	0.95 [0.79, 1.14]	0.573
Middle	1.02 [0.82, 1.27]	0.848	0.96 [0.76, 1.21]	0.724
Less poor	1.26 [1.01, 1.56]	0.039	1.18 [0.94, 1.48]	0.150
Least poor	1.46 [1.15, 1.85]	0.002	1.31 [1.03, 1.67]	0.026
Area of residence				
Rural	Reference		Reference	
Urban	0.90 [0.73, 1.12]	0.337	0.78 [0.62, 0.98]	0.030
Marital status				
Unmarried	Reference		Reference	
Currently married	1.09 [0.92, 1.28]	0.327	1.22 [1.02, 1.45]	0.027
Employment status				
Unemployed	Reference		Reference	
Informally employed	0.90 [0.78, 1.04]	0.150	1.04 [0.90, 1.20]	0.624
Formally employed	0.96 [0.80, 1.16]	0.685	1.11 [0.93, 1.32]	0.259
Parity				
1 child	Reference		Reference	
2–3 children	0.72 [0.62, 0.84]	<0.001	0.75 [0.62, 0.89]	0.002
≥4 children	0.48 [0.40, 0.59]	<0.001	0.56 [0.43, 0.73]	<0.001
Insurance coverage				
Uninsured	Reference		Reference	
Insured	1.47 [1.22, 1.79]	<0.001	1.29 [1.06, 1.58]	0.012

a Reported odds ratios (OR) compare the odds of receiving early ANC (first ANC visit in the first trimester of pregnancy) vs receiving no or delayed ANC.

b Adjusted odds ratio (aOR) is adjusted for child’s year of birth and all other variables reported in the table.

There did not appear to be general population-wide change over time in early ANC initiation after the voucher programme was fully implemented in Period 2 (aOR = 1.23; 95% CI: 0.51–2.97) or after free maternity services were introduced in Period 3 (aOR = 1.08; 95% CI: 0.61–1.91). However, the interaction term for intervention group and Period 3 suggests that voucher counties may have experienced a marginally higher increase in early ANC initiation than comparison counties after free maternity services were introduced (aOR = 1.35; 95% CI: 0.95–1.93; Table 4).

### Use of maternal care across the continuum

The proportion of births with discontinuous care across the maternal health continuum decreased from approximately 52% of 1+ ANC users in both study groups in Period 1 to 23.0% and 30.3% of 1+ ANC users in Period 3 in voucher and comparison counties, respectively (Table 3). Over the same periods, the proportion of births that received continuous care and completed the maternal health continuum pathway as recommended increased from 31.7% to 56.1% in voucher counties and 30.6% to 46.8% in comparison counties. In both study groups, the use of continuous, suboptimal care remained fairly constant over time, ranging from 16.3% in voucher counties in Period 1 to 22.9% in comparison counties in Period 3.

**Figure 2.**
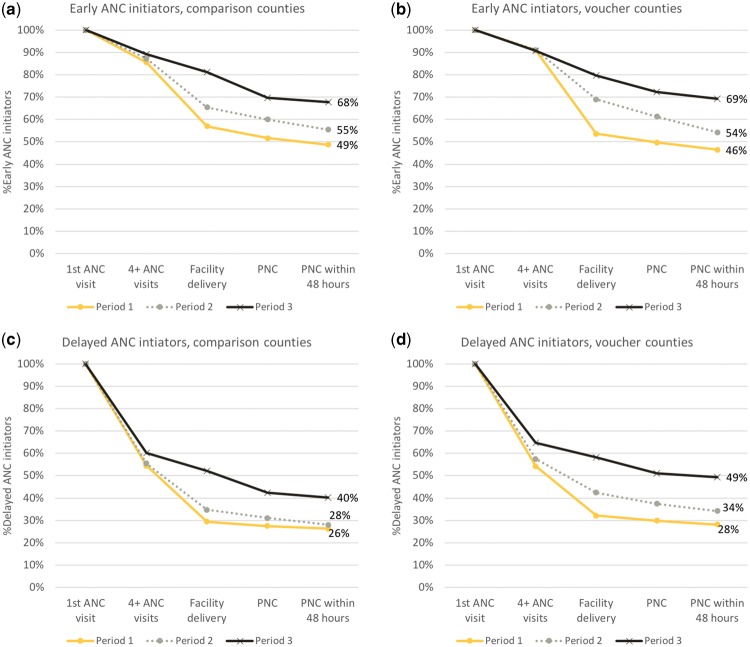
Cumulative survival in continuum of care pathway among ANC users over time.

To understand the importance of early ANC initiation, Figure 2 illustrates the retention, or cumulative survival, of 1+ ANC users through the maternal health continuum over time, by intervention group and timing of first ANC visit. In both voucher and comparison counties, the percentage of early ANC users who completed the maternal health continuum as recommended (receiving 4+ ANC visits, facility delivery and PNC within 48 h) increased from nearly 50% in Period 1 to approximately 70% in Period 3, after free maternity services were introduced (Figure 2a and b). Delayed ANC initiators appeared much less likely than early initiators to complete the maternal health pathway as recommended, with <30% of all births completing the pathway in Period 1, to 49% of births in voucher counties and 40% of births in comparison counties completing the pathway in Period 3 (Figure 2c and d). Among delayed ANC users, the steepest drop-off in the continuum of care occurred between 1+ and 4+ ANC visits, while early ANC initiators experienced the steepest drop-off between 4+ ANC visits and facility delivery.

#### Continuous care (suboptimal + completed pathway) vs discontinuous care

Both timing and source of ANC were associated with improved continuity of care among ANC users (Table 5). We found that women with early ANC initiation had 1.32 times higher adjusted odds of receiving continuous care, or contact with the health system at each point in the continuum from ANC to facility delivery to PNC, compared with women who started ANC after their first trimester (95% CI: 1.13–1.55). Additionally, women who obtained ANC in the private sector had nearly two times greater odds of receiving continuous care compared with those who received care in the public sector (aOR = 1.93; 95% CI: 1.45–2.55).

**Table 5.** Model 2—determinants of receiving continuous care among ANC users (*n* = 6990).

	Unadjusted		Adjusted^b^	
	OR^a^ [95% CI]	Wald test (*P*-value)	aOR^a^ [95% CI]	Wald test (*P*-value)
Intervention group				
Comparison county	Reference		Reference	
Voucher county	1.22 [0.90, 1.64]	0.198	1.50 [1.08, 2.11]	0.018
Period				
Period 2 (base = Period 1)	1.43 [1.27, 1.60]	<0.001	4.00 [1.89, 8.44]	<0.001
Period 3 (base = Period 2)	2.14 [1.84, 2.49]	<0.001	1.21 [0.71, 2.07]	0.467
Interaction terms				
Period 2 × Voucher county	1.45 [1.17, 1.79]	0.001	1.33 [1.06, 1.67]	0.014
Period 3 × Voucher county	1.01 [0.75, 1.36]	0.956	1.02 [0.75, 1.41]	0.855
ANC timing				
Delayed ANC	Reference		Reference	
Early ANC	1.63 [1.41, 1.90]	<0.001	1.32 [1.13, 1.55]	0.001
Source of ANC				
Public sector or home/other	Reference		Reference	
Private sector	2.04 [1.48, 2.82]	<0.001	1.93 [1.45, 2.55]	<0.001
Maternal age at birth				
<25 years	Reference		Reference	
25–34 years	0.85 [0.74, 0.97]	0.021	1.25 [1.09, 1.43]	0.002
≥35 years	0.58 [0.49, 0.68]	<0.001	1.35 [1.08, 1.70]	0.011
Highest level of education				
No education and incomplete primary	Reference		Reference	
Completed primary and incomplete secondary	1.84 [1.57, 2.15]	<0.001	1.54 [1.33, 1.78]	<0.001
Completed secondary/higher	4.42 [3.56, 5.49]	<0.001	2.67 [2.17, 3.28]	<0.001
Wealth quintile				
Poorest	Reference		Reference	
Poorer	1.18 [0.95, 1.47]	0.141	1.13 [0.90, 1.41]	0.277
Middle	1.34 [1.06, 1.71]	0.016	1.12 [0.88, 1.42]	0.361
Less poor	1.96 [1.50, 2.56]	<0.001	1.46 [1.10, 1.92]	0.008
Least poor	2.26 [1.73, 2.95]	<0.001	1.38 [1.07, 1.79]	0.014
Area of residence				
Rural	Reference		Reference	
Urban	1.27 [0.90, 1.80]	0.171	1.11 [0.83, 1.56]	0.483
Marital status				
Unmarried	Reference		Reference	
Currently married	0.88 [0.77, 1.01]	0.067	1.06 [0.91, 1.25]	0.452
Employment status				
Unemployed	Reference		Reference	
Informally employed	1.03 [0.88, 1.21]	0.735	1.32 [1.11, 1.56]	0.002
Formally employed	1.05 [0.87, 1.27]	0.629	1.37 [1.11, 1.71]	0.005
Parity				
1 child	Reference		Reference	
2–3 children	0.64 [0.54, 0.76]	<0.001	0.67 [0.56, 0.82]	<0.001
≥4 children	0.28 [0.23, 0.34]	<0.001	0.31 [0.24, 0.40]	<0.001
Insurance coverage				
Uninsured	Reference		Reference	
Insured	2.96 [2.39, 3.67]	<0.001	1.96 [1.58, 2.44]	<0.001

a Reported odds ratios (OR) compare the odds of receiving continuous care (sub-optimal care and completed pathway) vs discontinuous care.

b Adjusted odds ratio (aOR) is adjusted for child’s year of birth and all other variables reported in the table.

Higher educational attainment appears to have a strong association with continuity of care; the adjusted odds of continuous care use were 1.54 times higher (95% CI: 1.33–1.78) among births to women who completed primary education and 2.67 times higher (95% CI: 2.17–3.28) among births to women with secondary or higher education compared with those educated below the primary level. Other socio-economic factors such as higher maternal age, belonging to the less and least poor wealth quintiles and being informally or formally employed were also associated with higher use of continuous care among ANC users. Additionally, health insurance coverage was associated with nearly two times greater odds of receiving continuous care (aOR = 1.96; 95% CI: 1.58–2.44). Higher parity was the only factor negatively associated with continuity of care; ANC users with two to three children and four or more children had 33% (aOR = 0.67; 95% CI: 0.56–0.82) and 69% (aOR = 0.31; 95% CI: 0.24–0.31) lower odds of receiving continuous care compared with those with only one birth.

There was a 4-fold increase in the odds of continuous care use among ANC users in both voucher and comparison counties between the pre-intervention/rollout phase of the voucher programme in Period 1 to the full implementation phase in Period 2 (aOR = 4.00; 95% CI: 1.89–8.44). Overall, the adjusted odds of continuous care use were 1.50 times higher in voucher counties than in comparison counties (95% CI: 1.08–2.11). In addition to the generally higher use of continuous care in voucher counties, there was a positive interaction between intervention group and Period 2. This suggests that the implementation of the voucher programme resulted in a greater increase over time in the odds of continuous care use in voucher counties than that observed in comparison counties (aOR = 1.33; 95% CI: 1.06–1.67).

#### Continuous, completed pathway vs continuous, suboptimal care

Among users of continuous care, the adjusted odds of completing the maternal health pathway as recommended (receiving 4+ ANC, facility delivery and PNC within 48 h of delivery) were 3.80 times greater (95% CI: 3.08–4.69) among early ANC initiators compared with late initiators (Table 6). Compared with continuous care users who received services exclusively in the public sector, users of all private services (aOR = 1.02; 95% CI: 0.84–1.24) and a mix of public and private services (aOR = 1.01; 95% CI: 0.80–1.26) appeared to have similar odds of completing the maternal healthcare pathway as recommended. Relative to continuous care users younger than 25 years, women aged 25–34 years and above 35 years had 1.37 (95% CI: 1.12–1.67) and 1.58 (95% CI: 1.18–2.11) times higher adjusted odds of completing the maternal health pathway as recommended, respectively. Other factors associated with higher completion of the maternal health continuum included completing secondary or higher education (aOR = 1.42; 95% CI: 1.13–1.78), being currently married (aOR = 1.30; 95% CI: 1.04–1.61), and having health insurance coverage (aOR = 1.30; 95% CI: 1.03–1.64). Having higher parity was associated with lower odds of completing the pathway; births to women with two to three children had 24% lower odds (aOR = 0.76; 95% CI: 0.60–0.95) of completing the pathway, and births to women with four or more children had 36% lower odds (aOR = 0.64; 95% CI: 0.48–0.86) of completing the pathway as recommended.

**Table 6:** Model 3—determinants of completing maternal health pathway among continuous care users (*n* = 3802).

	Unadjusted		Adjusted^b^	
	OR^a^ [95% CI]	Wald test (*P*-value)	aOR^a^ [95% CI]	Wald test (*P*-value)
Intervention group				
Comparison county	Reference		Reference	
Voucher county	1.09 [0.94, 1.25]	0.247	1.02 [0.79, 1.32]	0.880
Period				
Period 2 (base = Period 1)	0.95 [0.80, 1.11]	0.489	0.85 [0.25, 2.85]	0.785
Period 3 (base = Period 2)	1.35 [1.08, 1.68]	0.008	1.02 [0.56, 1.87]	0.944
Interaction terms				
Period 2 × Voucher county	0.88 [0.64, 1.20]	0.408	0.82 [0.59, 1.15]	0.254
Period 3 × Voucher county	1.38 [0.89, 2.13]	0.143	1.19 [0.77, 1.87]	0.419
ANC timing				
Delayed ANC	Reference		Reference	
Early ANC	3.89 [3.17, 4.76]	<0.001	3.80 [3.08, 4.69]	<0.001
Source of continuous care				
All services public sector	Reference		Reference	
All services private sector	1.09 [0.91, 1.30]	0.336	1.02 [0.84, 1.24]	0.850
Mixed public and private sector	1.09 [0.88, 1.34]	0.427	1.01 [0.80, 1.26]	0.947
Maternal age at birth				
<25 years	Reference		Reference	
25–34 years	1.16 [0.98, 1.37]	0.092	1.37 [1.12, 1.67]	0.002
≥35 years	1.07 [0.86, 1.35]	0.531	1.58 [1.18, 2.11]	0.003
Highest level of education				
No education and incomplete primary	Reference		Reference	
Completed primary and incomplete secondary	1.05 [0.87, 1.27]	0.580	1.05 [0.85, 1.28]	0.674
Completed secondary/higher	1.65 [1.33, 2.03]	<0.001	1.42 [1.13, 1.78]	0.003
Wealth quintile				
Poorest	Reference		Reference	
Poorer	0.87 [0.67, 1.12]	0.267	0.87 [0.67, 1.13]	0.288
Middle	1.02 [0.79, 1.31]	0.893	1.00 [0.78, 1.29]	0.985
Less poor	0.98 [0.78, 1.23]	0.870	0.87 [0.70, 1.08]	0.210
Least poor	1.18 [0.94, 1.47]	0.154	0.96 [0.76, 1.22]	0.757
Area of residence				
Rural	Reference		Reference	
Urban	1.00 [0.85, 1.17]	0.982	1.00 [0.82, 1.20]	0.967
Marital status				
Unmarried	Reference		Reference	
Currently married	1.24 [1.03, 1.50]	0.026	1.30 [1.04, 1.61]	0.021
Employment status				
Unemployed	Reference		Reference	
Informally employed	0.90 [0.78, 1.05]	0.188	0.93 [0.60, 1.11]	0.427
Formally employed	1.03 [0.84, 1.26]	0.789	1.04 [0.83, 1.30]	0.714
Parity				
1 child	Reference		Reference	
2–3 children	0.81 [0.68, 0.97]	0.022	0.76 [0.60, 0.95]	0.016
≥4 children	0.73 [0.59, 0.89]	0.002	0.64 [0.48, 0.86]	0.003
Insurance coverage				
Uninsured	Reference		Reference	
Insured	1.57 [1.29, 1.91]	<0.001	1.30 [1.03, 1.64]	0.028

a Reported odds ratios (OR) compare the odds of completing the maternal health pathway vs receiving continuous, sub-optimal care.

b Adjusted odds ratio (aOR) is adjusted for child’s year of birth and all other variables reported in the table.

There does not appear to be general change over time completion of the maternal health pathway as recommended among users of continuous care at the start of Period 2 (aOR = 0.0.85; 95% CI: 0.25–0.85) or Period 3 (aOR = 1.02; 95% CI: 0.56–1.87). Additionally the voucher programme did not appear to have any additional impact on completion of the maternal healthcare pathway as recommended after full implementation of the programme in Period 2 (interaction term aOR: 0.82; 95% CI: 0.59–1.15) or introduction of the free maternity services policy in Period 3 (interaction term aOR: 1.02; 95% CI: 0.56–1.87).

Table 7 presents a summary of the results of the three regression models examining determinants of early ANC initiation, continuous care use and completion of the maternal healthcare pathway as recommended.

**Table 7.** Summary of the effects of determinants on use of care across the maternal health continuum.

	Model 1: early ANC	Model 2: continuous care	Model 3: complete maternal healthcare pathway
Intervention group			
Voucher county (vs comparison)	None	Positive	None
Period			
Intro of voucher programme (Period 2 vs Period 1)	None	Positive	None
Intro of free maternity services policy (Period 3 vs 2)	None	None	None
Interaction terms			
Intro of voucher programme × Voucher county	None	Positive	None
Intro of free maternity services × Voucher county	Positive	None	None
Sociodemographic characteristics			
Higher maternal age at birth	Negative	Positive	Positive
Higher educational attainment	None	Positive	Positive
Higher wealth quintile	Positive	Positive	None
Urban residence	Negative	None	None
Marriage	Positive	None	Positive
Formal or informal employment	None	Positive	None
Higher parity	Negative	Negative	Negative
Health insurance coverage			
Insured	Positive	Positive	Positive
Pregnancy care			
Early ANC initiation	NA	Positive	Positive
Use of private sector ANC	NA	Positive	NA
Use of private/mixed continuous care	NA	NA	None

Positive effect (positive), *P* < 0.10.

Negative effect (negative), *P* < 0.10.

No effect (none), *P* > 0.10.

NA, not applicable.

## Discussion

Previous research on health financing for maternal health services has focused on the effect of financing interventions or policy changes on the use of services at individual points along the continuum from a woman’s pregnancy to the post-partum period, such as ANC, delivery care or PNC. Our study is unique in that it examines the population-level effects of subsidized vouchers and user fee removal on continuity of maternal care from a birth-centered perspective. Our findings show that prior to the implementation of the maternal health voucher programme and introduction of the free maternity services policy in Kenya, nearly all reported births in our study counties received at least one ANC visit. Despite this high contact with the health system during pregnancy, we found that after their initial ANC visit, a substantial proportion of women did not subsequently access health services across the maternal health continuum as recommended, with 4+ ANC visits, facility delivery and timely PNC. This research has important implications, particularly in light of results from a recent systematic review in low- and middle-income countries (LMICs) suggesting that strengthening the linkages between ANC, delivery care and PNC can lead to reductions in perinatal, neonatal and maternal mortality, even when recommendations regarding frequency of ANC and timing of ANC and PNC are not met (Kikuchi *et al.*, 2015).

Overall, our findings suggest that before the free maternity services policy was introduced, full implementation of the voucher programme improved use of continuous care among ANC users; however, it did not appear to impact early ANC initiation among all births or completion of the maternal health pathway as recommended among users of continuous care. In addition to this intervention effect, there was a general increase in use of continuous care among ANC users in both voucher and comparison counties that coincided with implementation of the voucher programme. The findings further suggest that after the free maternity services policy was introduced, voucher counties may have experienced a significantly higher increase in early ANC initiation among all births than that observed in comparison counties. After controlling for all other variables in the model, there did not appear to be a general effect of the free maternity services policy on use of early ANC among all births or on either of the measured continuum of care outcomes. Additionally, across time and intervention groups, health insurance coverage was consistently independently associated with earlier ANC initiation among all births, greater use of continuous care among ANC users, and higher likelihood of completing the maternal health pathway as recommended among continuous care users.

To maximize the health impact of future maternal health financing efforts in Kenya, it is important to consider the underlying mechanisms by which the observed effects were achieved. A study of nationally representative health facility exit interview data with ANC clients in Kenya found that women who believed that they had enough money to pay for delivery care were four times as likely to intend to deliver under the supervision of a skilled birth attendant (Nyongesa *et al.*, 2018). Another study on the continuum for maternal healthcare in Tanzania found that women who had to pay for ANC were less likely to deliver in a health facility (Mohan *et al.*, 2017). As purchase of a maternal health voucher required women to pay an up-front subsidized fee for four ANC visits, delivery care and PNC, we suspect that this may have encouraged women to develop birth preparedness plans earlier in their pregnancies and reduced the risk of women having insufficient funds to seek facility-based care for childbirth. This, in turn, may have facilitated improved continuity of care and possibly earlier ANC initiation. Similarly, although health insurance schemes vary, women are often aware about which services are covered prior to seeking care. In contrast, uncertainty around which services were included under the free maternity services policy and reports of women being required to pay out-of-pocket for services, supplies and laboratory tests may have contributed to delayed initiation of ANC and discontinuous maternal care among women without access to the voucher programme or health insurance (Pyone *et al.*, 2017; Tama *et al.*, 2017). Another key difference between the financing mechanisms of the voucher programme, free maternity services policy and health insurance is that vouchers and insurance coverage both allowed women to seek care in public and private facilities, while the user fee removal policy only applied to public facilities. By making private sector services more accessible, the voucher programme and health insurance coverage may have contributed to reducing women’s barriers to timely maternal health service initiation and improving continuity of care. It is therefore important to understand the aspects of private sector maternal care that women value most. Neither the voucher programme nor free maternity services were associated with improved completion of the maternal health pathway among users of continuous care, suggesting a need to better understand the barriers to receiving care as recommended among those who make contact with the health system for ANC, facility delivery and PNC.

Our study corroborates research from other LMIC settings indicating that women’s experiences during ANC are critical to their subsequent use of delivery and PNC services (Guliani *et al.*, 2012; Adjiwanou and LeGrand, 2013; Ensor *et al.*, 2014; Anastasi *et al.*, 2015; Wang and Hong, 2015; Owili *et al.*, 2016; Singh *et al.*, 2016; Chukwuma *et al.*, 2017). We found that starting ANC in the first trimester of pregnancy was associated with increased use of continuous care, or of making contact with health services at each point along the continuum from ANC to PNC. Additionally, given that early ANC initiators were more likely to receive 4+ ANC visits, starting ANC within the first trimester was also associated with greater completion of the maternal health pathway as recommended, with 4+ ANC visits, facility delivery and PNC within 48 h of delivery. Despite these strong associations between the timing of ANC initiation and effective use of maternal health services, fewer than 33% of women in both voucher and comparison counties started ANC within the first trimester throughout the study recall period. To facilitate further improvements in coverage of care across the maternal health continuum, policymakers in Kenya must therefore consider how to alleviate barriers to earlier ANC initiation, particularly focused on women who are older, poorer, unmarried, living in urban areas and with higher parity.

We also found that users of private sector ANC services in both voucher and comparison counties were nearly twice as likely to receive continuous care compared with those who received ANC in the public sector or at home. A recent analysis of data from 28 countries in sub-Saharan Africa found that women who received better content of ANC were more likely to have a skilled birth attendant (Chukwuma *et al.*, 2017). Another study of 23 countries in sub-Saharan Africa found that ANC quality of care was higher in private not-for-profit facilities than in the public sector and lower in private commercial facilities (Powell-Jackson *et al.*, 2015). Further, an analysis of exit interview data from a nationally representative health facility assessment in Kenya revealed that women who used private sector ANC reported higher client satisfaction scores compared with those who used public sector services (Do *et al.*, 2017). Our findings therefore indicate a need to investigate how differences in the quality of care offered by different providers might help explain the greater use of facility delivery and PNC services among private sector ANC users in Kenya.

With regards to sociodemographic determinants of how women use care across the maternal health continuum, higher parity was the only factor negatively associated with all three outcomes (early ANC initiation, continuous care and completing the maternal health pathway as recommended), meaning that it has a strong cumulative effect (Table 7). This finding is consistent with other studies on determinants of retention in the maternal care continuum, and suggests a need to consider how best to provide education on the importance of continuity of care and reduce barriers to seeking timely and continuous care in women’s second pregnancies and beyond (Guliani *et al.*, 2012; Ensor *et al.*, 2014; Anastasi *et al.*, 2015; Wang and Hong, 2015; Singh *et al.*, 2016; Chukwuma *et al.*, 2017; Mohan *et al.*, 2017). Although the effects and cumulative nature of socio-economic indicators such as educational attainment, wealth quintile and employment status varied, our findings suggest that none of the health financing interventions studied were sufficient to completely eliminate socio-economic disparities in timely initiation and continuity of maternal care.

This research has some limitations. The study sample was drawn from communities within 5 km of a health facility. Within these communities, poor women were purposively selected for inclusion. Additionally, where more than one eligible woman lived within a household, the youngest woman was selected to participate. This approach may have introduced biases to the sample that over-represent the experiences of women who live within closer proximity to health services and are younger and poorer than the general population. This sampling strategy also necessitates careful interpretation of the findings on wealth-related inequities. Assuming the purposive sampling was successful in identifying the poorest households in each community, the results reflect differences in access to care among the poor rather than between the wealthy and the poor. While the use of local administrators to help identify the poorest households may have also biased sampling, our analysis comparing the household assets in the voucher study sample to the general population suggests that compared with the national distribution of wealth in Kenya, our sample is poorer and the gap between the poorest and least poor wealth quintiles in our study is smaller (Supplementary data 2). Though the missing data in the 2016 survey is unlikely to impact our parameter estimates, as the data are missing at random and only missing in the outcome variables, it contributed to a reduced sample size (Dennis *et al.*, 2018). This may have impacted our ability to detect the effects of the free maternity services policy on our outcomes of interest.

Another limitation of this quasi-experimental study design is that we are attributing observed changes over time to the voucher programme and free maternity services policy; however, our findings may have also been affected by other programmes, policies and events in our study counties. For instance, since 2013, the Kenyan health system has experienced a number of challenges related to the decentralization of government and removal of user fees for maternal care, which are perceived to have contributed to reduced quality of care and unauthorized fees in some facilities (Nyikuri *et al.*, 2015; Barasa *et al.*, 2017; Kilonzo *et al.*, 2017; Tama *et al.*, 2017; Tsofa *et al.*, 2017). Additionally, concerns about salary delays, inadequate staffing and job insecurity led to multiple health worker strikes since the policy changes (Nyikuri *et al.*, 2015; Kilonzo *et al.*, 2017). All of these factors may have influenced our study counties in ways that are poorly documented and difficult to assess.

## Conclusions

Overall, our study illustrates the value of examining the way in which maternal health interventions affect how women use care across the continuum from pregnancy to the post-partum period and has important implications for maternal health financing in Kenya and similar settings. Although the reproductive health voucher programme and free maternity services policy contributed to high use of facility delivery services, we found that continuity of care remained subpar, with approximately one-quarter to one-third of ANC users receiving discontinuous or incomplete care (Dennis *et al.*, 2018). To maximize the benefits of maternal health financing interventions and polices in Kenya, it is therefore critical to better understand and address the non-financial mechanisms driving use of care across the maternal health continuum. The strong effect of using private sector ANC on subsequent use of facility delivery and PNC within 48 h suggests a need to further investigate the role of health providers and quality of care on ensuring linkages between the different stages of maternal care. Additionally, our findings that even within this population of poor women, those with lower parity and higher educational attainment, wealth and employment status were more likely to use continuous care indicate that health financing alone is insufficient for reducing inequities in use of care across the maternal health continuum.

## Supplementary Material

Supplementary Data 1

Supplementary Data 2
