# Supplementary material for: Initiation and continuity of maternal healthcare: examining the role of vouchers and user-fee removal on maternal health service use in Kenya
Source: Health Policy Plan. 2019 Mar 6;34(2):120–31. doi: 10.1093/heapol/czz004 (PMC6481282; doi:10.1093/heapol/czz004)
Supplement: Supplementary Data 1 [file czz004_supplementary_data_1.docx]

**Supplement 1: Description of women sampled**

|  | Period 1  (Pre-voucher/rollout period)  N=1,888 | | |  | Period 2  (Full voucher implementation)  N=2,198 | | |  | Period 3  (Free maternity services introduced)  N=1,237 | | |
| --- | --- | --- | --- | --- | --- | --- | --- | --- | --- | --- | --- |
|  | **Comparison counties** | **Voucher counties** | **p-value** |  | **Comparison counties** | **Voucher counties** | **p-value** |  | **Comparison counties** | **Voucher counties** | **p-value** |
| Age group (years) (%) |  |  | p=0.002 |  |  |  | p=0.079 |  |  |  | p=0.018 |
| 15-24 | 23.1 | 32.3 |  |  | 32.5 | 38.8 |  |  | 32.8 | 39.5 |  |
| 25-34 | 50.6 | 48.9 |  |  | 49.3 | 46.2 |  |  | 50.1 | 45.1 |  |
| 35+ | 26.3 | 18.9 |  |  | 18.3 | 15.0 |  |  | 17.1 | 15.6 |  |
| Educational attainment (%) |  |  | p=0.021 |  |  |  | p=0.351 |  |  |  | p=0.382 |
| Below primary | 26.2 | 32.2 |  |  | 28.1 | 32.6 |  |  | 24.3 | 27.6 |  |
| Completed primary | 58.1 | 55.3 |  |  | 53.6 | 51.2 |  |  | 51.5 | 47.4 |  |
| Completed secondary/higher | 19.7 | 12.5 |  |  | 18.3 | 16.2 |  |  | 24.2 | 25.0 |  |
| Wealth quintile (%) |  |  | p=0.089 |  |  |  | p=0.786 |  |  |  | p=0.505 |
| Poorest | 18.1 | 20.3 |  |  | 21.1 | 20.1 |  |  | 17.7 | 22.3 |  |
| Poorer | 19.6 | 21.7 |  |  | 22.4 | 20.6 |  |  | 22.8 | 20.0 |  |
| Middle | 22.2 | 20.9 |  |  | 19.0 | 18.8 |  |  | 19.1 | 19.2 |  |
| Less poor | 19.8 | 18.4 |  |  | 18.1 | 21.4 |  |  | 22.1 | 19.7 |  |
| Least poor | 20.3 | 18.7 |  |  | 19.4 | 19.1 |  |  | 18.2 | 18.8 |  |
| Residence |  |  | p=0.478 |  |  |  | p=0.365 |  |  |  | p=0.587 |
| Rural | 87.5 | 82.4 |  |  | 87.4 | 80.1 |  |  | 90.2 | 85.9 |  |
| Urban | 12.5 | 17.6 |  |  | 12.6 | 19.9 |  |  | 9.8 | 14.1 |  |
| Current marital status (%) |  |  | p=0.265 |  |  |  | p=0.014 |  |  |  | p=0.957 |
| Unmarried | 16.7 | 19.1 |  |  | 16.1 | 20.8 |  |  | 22.5 | 22.3 |  |
| Married/cohabiting | 83.3 | 80.9 |  |  | 83.9 | 79.2 |  |  | 77.5 | 77.7 |  |
| Woman’s employment (%) |  |  | p=0.453 |  |  |  | p=0.022 |  |  |  | p=0.140 |
| Unemployed | 34.6 | 39.2 |  |  | 40.4 | 50.4 |  |  | 45.4 | 51.3 |  |
| Informally employed | 43.6 | 41.1 |  |  | 48.0 | 39.1 |  |  | 48.1 | 39.8 |  |
| Formally employed | 21.8 | 19.7 |  |  | 11.5 | 10.6 |  |  | 6.4 | 8.8 |  |
| Parity (%) |  |  | p=0.451 |  |  |  | p=0.484 |  |  |  | p=0.978 |
| 1 child | 17.7 | 20.5 |  |  | 21.1 | 23.6 |  |  | 27.9 | 27.3 |  |
| 2-3 children | 44.2 | 43.4 |  |  | 43.6 | 43.0 |  |  | 44.6 | 45.0 |  |
| ≥4 children | 38.1 | 36.2 |  |  | 35.3 | 33.4 |  |  | 27.5 | 27.8 |  |
| Health insurance enrollment (%) |  |  | p<0.001 |  |  |  | p=0.032 |  |  |  | p=0.283 |
| Uninsured | 86.5 | 93.4 |  |  | 86.3 | 90.8 |  |  | 79.7 | 82.8 |  |
| Insured | 13.5 | 6.6 |  |  | 13.7 | 9.2 |  |  | 20.3 | 17.2 |  |
| Total no. women | **871** | **1017** |  |  | **1066** | **1132** |  |  | **592** | **645** |  |
