# Supplementary material for: Initiation and continuity of maternal healthcare: examining the role of vouchers and user-fee removal on maternal health service use in Kenya
Source: Health Policy Plan. 2019 Mar 6;34(2):120–31. doi: 10.1093/heapol/czz004 (PMC6481282; doi:10.1093/heapol/czz004)
Supplement: Supplementary Data 2 [file czz004_supplementary_data_2.docx]

**Supplement 2**

**Table 1:** Comparison of select household assets by wealth quintile in voucher surveys vs. 2014 Kenya DHS

We compared the voucher study questionnaires to the indicators comprising the EquityTool^1^, a resource for comparing the wealth of survey respondents to the wealth of the national population. The voucher surveys only collected information on 8 of the 13 household assets required to use the EquityTool; although we could not run the tool, we compared the distribution of these 8 assets between the 2014 Kenya DHS sample and the voucher survey samples.

|  | **Q1**  **(Poorest)** | | **Q2** | | **Q3** | | **Q4** | | **Q5**  **(Least poor)** | |
| --- | --- | --- | --- | --- | --- | --- | --- | --- | --- | --- |
|  | **KDHS 2014** | **Voucher surveys** | **KDHS 2014** | **Voucher surveys** | **KDHS 2014** | **Voucher surveys** | **KDHS 2014** | **Voucher surveys** | **KDHS 2014** | **Voucher surveys** |
| ***%Households with:*** |  |  |  |  |  |  |  |  |  |  |
| electricity | 0.2% | 1.3% | 0.9% | 5.1% | 7.5% | 10.7% | 45.7% | 25.3% | 95.3% | 49.9% |
| television | 0.6% | 1.6% | 2.2% | 7.6% | 12.4% | 17.5% | 50.4% | 28.1% | 96.1% | 49.4% |
| radio | 32.9% | 51.3% | 58.2% | 63.6% | 74.6% | 70.2% | 79.7% | 75.8% | 87.0% | 79.8% |
| *floor type:* |  |  |  |  |  |  |  |  |  |  |
| cement | 1.8% | 0.6% | 8.0% | 11.1% | 34.8% | 23.6% | 76.6% | 52.7% | 75.1% | 90.2% |
| earth or sand | 72.5% | 99.0% | 51.4% | 88.3% | 33.1% | 75.1% | 11.7% | 45.6% | 0.4% | 7.5% |
| other | 25.7% | 0.5% | 40.6% | 0.6% | 32.0% | 1.2% | 11.7% | 1.7% | 24.5% | 2.4% |
| *external wall type:* |  |  |  |  |  |  |  |  |  |  |
| dung, mud, sod | 58.4% | 73.1% | 63.7% | 51.1% | 44.3% | 36.8% | 13.5% | 24.6% | 0.6% | 7.7% |
| other | 41.6% | 26.9% | 36.3% | 48.9% | 55.7% | 63.2% | 86.5% | 75.4% | 99.4% | 92.4% |
| *roof type:* |  |  |  |  |  |  |  |  |  |  |
| thatch, grass, makuti | 53.5% | 32.7% | 9.3% | 4.2% | 1.6% | 0.9% | 0.2% | 0.5% | 0.2% | 0.5% |
| other | 46.5% | 67.4% | 90.7% | 95.8% | 98.4% | 99.2% | 99.8% | 99.5% | 99.9% | 99.5% |
| *main cooking fuel:* |  |  |  |  |  |  |  |  |  |  |
| wood | 95.6% | 59.8% | 90.2% | 58.6% | 79.3% | 52.5% | 47.2% | 36.1% | 9.5% | 22.6% |
| LPG/natural gas | 0.0% | 0.0% | 0.0% | 0.2% | 0.2% | 0.3% | 3.0% | 0.8% | 42.7% | 4.3% |
| other | 4.4% | 40.2% | 9.8% | 41.2% | 20.6% | 47.3% | 49.8% | 63.1% | 47.8% | 73.1% |
| *toilet type:* |  |  |  |  |  |  |  |  |  |  |
| no facility, bush, field | 50.8% | 20.5% | 7.3% | 9.0% | 1.8% | 3.6% | 0.4% | 1.1% | 0.0% | 0.9% |
| other | 49.2% | 79.5% | 92.7% | 91.0% | 98.2% | 96.4% | 99.6% | 98.9% | 100.0% | 99.1% |

^1^Metrics for Management, (2018). The EquityTool. [online] Available at: http://www.equitytool.org/the-equity-tool-2/ [Accessed 14 Nov. 2018]

**Figure 1:** Distribution of wealth index scores by wealth quintile in the voucher study surveys

We examined the distribution of wealth index scores by wealth quintile using box plots. This shows that there is a gradient in asset ownership between our five wealth quintiles rather than a clear threshold between poorest and least poor.
